# Protective effects of glucocorticoid receptor antagonist Mifepristone on fear memory extinction impairment in a rat model of PTSD

**DOI:** 10.1192/j.eurpsy.2022.630

**Published:** 2022-09-01

**Authors:** Y.-P. Liu, C.-C. Lin

**Affiliations:** 1 Cheng Hsin General Hospital, Psychiatry, Taipei, Taiwan; 2 Academia Sinica, Genomics Research Center, Taipei, Taiwan

**Keywords:** extinction, fear memory, glucocorticoid receptor, prevention

## Abstract

**Introduction:**

Central glucocorticoid receptor (GR) has been found to play an important role in the interpretation of cognitive abnormalities of posttraumatic stress disorder (PTSD), particularly focused on the extinction failure of fear memory. Potential of using GR antagonist as a pharmacological agent to prevent PTSD-related fear memory disruption is worth investigating.

**Objectives:**

We aimed to examine whether GR antagonist Mifepristone (RU486) administered before single prolonged stress (SPS) can prevent rats from fear memory extinction impairment.

**Methods:**

In the present study, SPS was employed in rats to induce a rodent model of PTSD. 60 minutes before SPS, RU486 (20 mg/kg) was administered by intraperitoneal injection. Seven days after SPS, rats received a protocol of behavioral testing to measure their abilities of specific fear memory (by a cue-dependent fear conditioning paradigm) and nonspecific spatial memory (by T-maze). Neurochemically, we measured plasma corticosterone with or without dexamethasone suppression, activation ratio of GR and levels of norepinephrine, dopamine, and serotonin in amygdala, paraventricular nucleus, dorsal and ventral hippocampus.

**Results:**

Our results found that RU486 exerted protective effects on SPS-induced fear extinction impairment. Corticosterone of SPS-RU486 rats was less suppressed by dexamethasone. GR became less activated in dorsal hippocampus of SPS-RU486 rats.

**Conclusions:**

The findings supported the utility of GR antagonism in preventing the development of PTSD.

**Disclosure:**

No significant relationships.

